# More than translation: an exploratory case study of culturally-responsive modifications in digital mental health for multilingual families

**DOI:** 10.1186/s12913-026-15220-8

**Published:** 2026-07-30

**Authors:** Gabriella Bobadilla, Naomi Quezada, Erika L. Gustafson, Colleen Stiles-Shields

**Affiliations:** Institute for Juvenile Research, Department of Psychiatry, University of Illinois Chicago, 1747 W. Roosevelt Rd., Chicago, IL 60608 USA

**Keywords:** Multilingual families, Digital Low-Intensity Treatments (D-LITs), Modifications, Equity, Teen, Culturally-responsive, Exploratory case study

## Abstract

**Background:**

Approximately 68 million people in the U.S. speak a language other than English at home. Multilingual families face many barriers to accessing mental health care, including language and cultural differences. Digital low-intensity treatments (D-LITs) can potentially address these barriers by providing accessible, scalable interventions. However, D-LITs must be modified to account for the daily, real-world complexities of youth and implementation contexts. The current case study aimed to systematically describe modifications, informed by feedback from key players, to a monolingual D-LIT to increase access to evidence-based interventions for multilingual families (i.e., Spanish/English).

**Methods:**

The Teen Assess, Check, and Heal (TeACH) System is a D-LIT for teens with anxiety implemented via a randomized controlled trial across multiple pediatric primary care clinics serving patients in Chicago, Illinois, USA. By evaluating participant-facing materials and recruitment procedures during the design and implementation stages, we engaged in an iterative refinement process to improve the TeACH System to better serve youth with Spanish-speaking caregivers. Modifications were retroactively coded to document their context and purpose using the FRAME-IS, a framework for documenting modifications to the implementation strategies of evidence-based interventions.

**Results:**

Forty-two percent (155/366) of the teens who assented opted to include parental permission, and 14.6% indicated that Spanish was their guardian’s preferred language. Six key modifications aimed to (1) increase health equity through culturally-appropriate content; (2) improve fidelity to IRB consent procedures when engaging with Spanish-speaking caregivers; (3) increase reach of the TeACH System; and (4) improve feasibility of recruitment in primary care settings. Most of the modifications occurred during the implementation phase (83%) and were unplanned/reactive (66%). Recruitment staff led most (83%) of the decision-making regarding modifications. All modifications were applied across the intervention to improve TeACH’s ability to better serve multilingual families.

**Conclusion:**

Culturally-responsive modifications must be made at all stages of design and dissemination to ensure that research participation is equitable, even when an intervention is intended to be delivered in English. Culturally adapted and appropriate D-LITs may serve as a catalyst for enhancing the accessibility and utilization of effective digital tools to address mental health disparities experienced by multilingual families.

**Clinical trial number:**

Not applicable.

**Supplementary Information:**

The online version contains supplementary material available at https://doi.org/10.1186/s12913-026-15220-8.

## Background

Youth mental health has become an increasingly global priority [1, 2]. In the United States (U.S.), specifically, a significant rise in mental health challenges among youth [3] prompted a formal declaration of a youth mental health crisis in 2021 [4]. Despite this declaration, access to timely, evidence-based mental health care remains limited for many Americans due to several intersecting cultural, economic, and systemic barriers. Youth face unique challenges and experiences that contribute to the difficulties of accessing mental health services, including stigma, cost, complications in obtaining referrals, parental permissions/consent, and extensive waiting periods to connect with mental health providers [5, 6]. These barriers are even more pronounced for youth living in systemically-excluded communities, where structural inequities—such as socioeconomic disparities, under-resourced healthcare systems, and heightened exposure to childhood adversity–further hinder access to services [7, 8].

These structural barriers are further compounded for youth in multilingual families, who must also navigate linguistic and cultural incongruences within predominantly English-based systems of care. In this study, multilingual families were defined as those in which two or more languages are spoken at home, often including at least one language other than English. In the U.S., approximately 11.8 million youth (ages 5–17) and 46.4 million adults are multilingual [9, 10]. Within multilingual families, it is common for caregivers to have limited English proficiency—an estimated 26.3 million individuals nationwide—while youth in these families are often bilingual or English-dominant [11–13]. Multilingualism is associated with distinct strengths and challenges. At the individual level, multilingualism is associated with higher executive functioning and a reduced risk of cognitive decline [14]. However, at the family level, differences in language fluency between caregivers and youth can pose a range of communication-related challenges when navigating health services.

One major challenge in serving multilingual families is the limited access to professional interpreters and bilingual healthcare providers [11, 12, 15]. In the absence of formal language support, youth are often placed in the position of acting as ad hoc interpreters for their caregivers, which can compromise both the accuracy and confidentiality of clinical interactions [16]. This dynamic frequently leads to suppressed disclosure of symptoms—especially those related to psychological distress—which can ultimately contribute to inaccurate mental health evaluations [16]. As a result, clinicians may struggle to capture meaningful diagnostic information, particularly regarding mental health, where nuanced communication is essential. Even when a diagnosis is confirmed, multilingual families often face continued barriers in understanding medical and/or care instructions [11]. Together, these complex barriers contribute to reduced access and lower-quality care.

As such, an exploratory case study was conducted to characterize modifications made to a monolingual digital mental health (DMH) to increase access to evidence-based resources for multilingual families (i.e., Spanish/English) within Pediatric Primary Care (PPC). Consistent with the use of case studies in social sciences [17], the current study presents findings through a narrative of the linguistic, cultural, and contextual needs of the community in the implementation setting. The purpose of the present study was to emphasize the vital role of key players (i.e., recruitment staff, research participants) in informing more equitable and responsive deployment of DMH interventions for linguistically and culturally diverse families.

### Bridging the gap: Digital Low-Intensity Treatments (D-LITs)

DMH tools—interventions delivered via mobile apps, tablets, and other virtual/computerized platforms—are promising approaches to bridge the gap between multilingual families and mental health services [18–20]. Within the broader category of DMH, a subset of interventions are called digital low-intensity treatments (D-LITs). D-LITs can provide brief, scalable, evidence-based interventions that can be delivered digitally, often without requiring extensive clinical oversight [21, 22]. D-LITs have been deployed in multiple contexts to support early intervention and prevention efforts for anxiety, depression, and stress management among youth [21, 23]. They appeal to individuals who prefer to self-manage symptoms and may not otherwise seek traditional treatment [18]. Moreover, their digital format enables rapid delivery at the point of need and the potential to reach larger, more diverse populations often underrepresented in clinical care and research [18, 24]. Despite the growing promise of D-LITs—and DMH tools broadly—they are often developed, tested, and deployed primarily in English. This monolingual approach limits the accessibility and effectiveness of these interventions for youth and families from linguistically and culturally diverse backgrounds. By failing to address linguistic and cultural differences, digital interventions may inadvertently perpetuate existing inequities. Therefore, we need to better understand how D-LITs can be modified to meet the needs of multilingual families, particularly in real-world care settings where many available tools are still delivered only in English.

### A D-LIT for teens with anxiety: The Teen Assess, Check, and Heal (TeACH) system

The Teen Assess, Check, and Heal (TeACH) System is a D-LIT for teens, ages 13–17, that was developed in English using human-centered design methods [25]. The TeACH System consists of a brief anxiety screener, symptom feedback, and digital evidence-based resources. The TeACH System was implemented and assessed across multiple PPC clinics in Chicago, Illinois, USA. These PPC clinics are affiliated with two major academic hospital systems [MASKED]. Their primary campuses are located on the West Side of Chicago and serve diverse patient populations [26]. Many of the communities surrounding these hospitals have among the highest concentrations of Hispanic and Latiné residents in Chicago, with some neighborhoods where over 60–80% of the population identifies as Hispanic/Latiné [27]. Recent Census data indicate that approximately 34.7% of households in Chicago speak a language other than English, with 22.7% reporting Spanish as the language spoken at home [28]. While most teens receiving care in PPC settings speak English, both hospital systems serve a high number of multilingual families, particularly those who speak Spanish at home. Although the TeACH System was delivered in English, we recognized the unique needs of this population. As such, across the design, pre-implementation, and implementation stages of the TeACH System, we monitored patient-facing materials and recruitment procedures for accessibility with the local population [29]. The current study reports *modifications* to the TeACH System that focus on language, cultural context, and community needs to ensure its accessibility to multilingual teens with Spanish-speaking caregivers. The current study does not involve human subjects research, as the data consisted of modifications documented during team meetings for the randomized controlled trial evaluating the TeACH System [25].

### The science of modifications

Modifications are ad hoc or planned changes to evidence-based treatments (EBTs) or their delivery to improve fit, engagement, or effectiveness [30–32]. In implementation science, such modifications are recognized not as exceptions but as the norm for implementing EBTs in real-world settings [33]. This iterative refinement process reflects the need to align interventions with the realities of both the target population (e.g., teens from multilingual families) and the specific service contexts (e.g., PPC clinics) [34]. The well-documented 17-year lag in translating research evidence into clinical practice underscores the urgent need to understand the modifications required to ensure EBTs are both effective and practical in real-world settings [35]. As such, it is necessary to systematically account for and evaluate these modifications to inform ongoing development, evolution, and scaling of EBTs [34, 36]. Guided by the Framework for Reporting Adaptations and Modifications to Evidence-based Implementation Strategies (FRAME-IS)—a structured framework for mapping modifications to implementation strategies—we documented and analyzed the modifications made to the TeACH System during its pre-implementation and implementation phases [31, 37].

## Methods

### Application of FRAME-IS

**Fig. 1:**
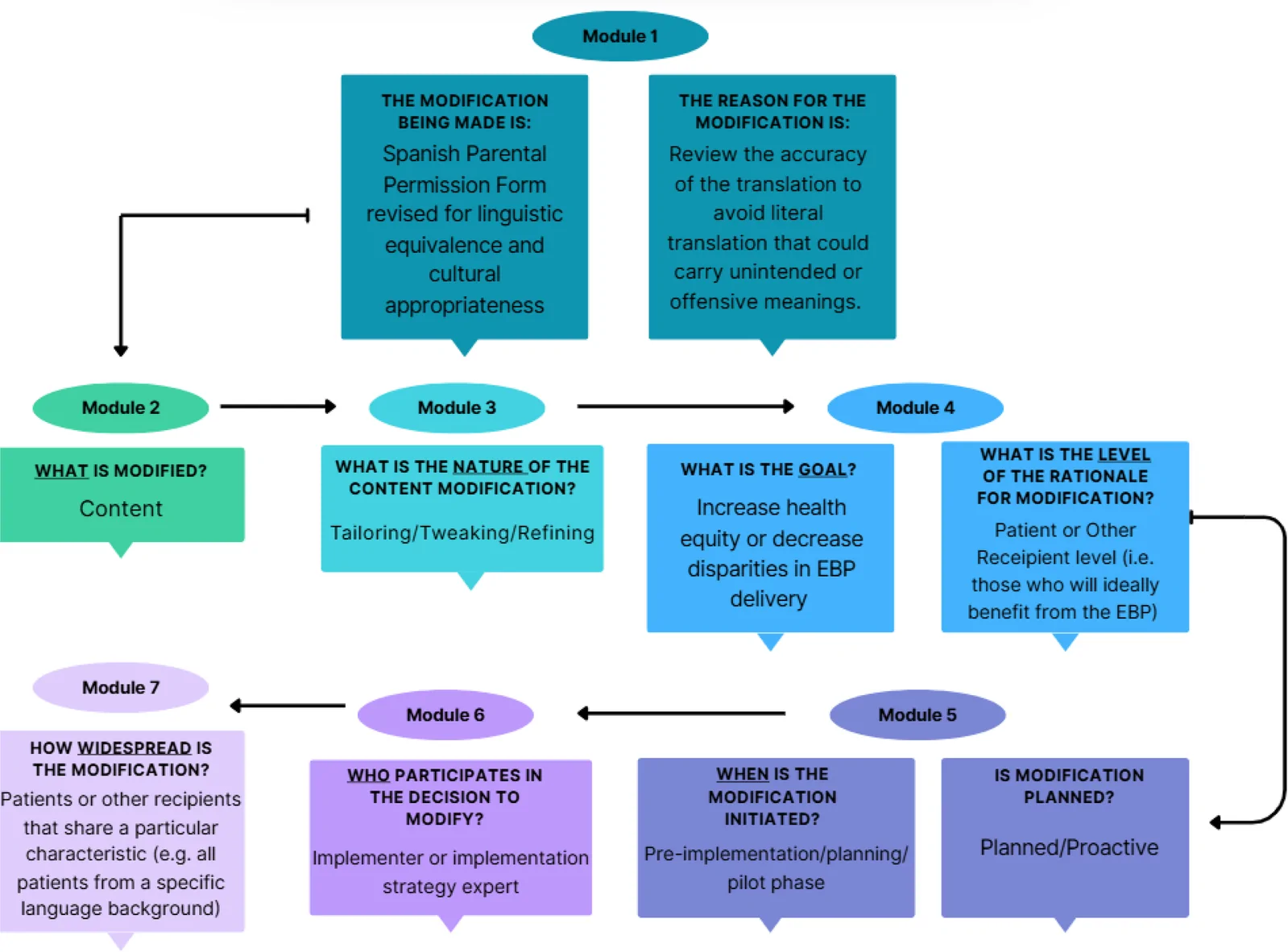
Example of FRAME-IS coding for the Spanish parental permission form modification

Using a descriptive observational design, this exploratory case study examined the iterative refinement process aimed at improving the TeACH System to better engage youth from multilingual families, particularly those with Spanish-speaking caregivers [17, 38]. Several implementation determinants were identified through the recruitment team’s observations. Documented modifications were retroactively coded using the FRAME-IS, providing a structured framework for mapping changes to implementation strategies for evidence-based practices, which included identifying their context and purpose [37]. The research team created an Excel spreadsheet that captured: (1) the modification being made and the reason for the modification; (2) modification category (e.g., content, training, context); (3) nature of the modification (e.g., removing/adding/refining strategies); (4) goal of the modification and at what level (e.g., participant, clinician, staff); (5) when the modification was made (pre-implementation, implementation) and whether it was planned; (6) who was involved in the decision; and (7) scope of the modification [37, 39]. The research team extracted data from meeting minutes and summaries of documented modifications. The data were entered into a matrix outlined by the seven core FRAME-IS modules. Figure 1 illustrates an example of using the FRAME-IS modules to code one of our modifications.

Once all data were entered into the matrix, the research team met to discuss the initial coding of modifications to the TeACH System using the FRAME-IS modules, aiming to accurately capture each modification. Upon reaching consensus, the research team reviewed the matrix to identify similarities and differences between the modifications.

## Results

### TeACH system trial participant characteristics

In the trial, a total of 366 positive assents were obtained. A total of 239 participants completed the demographics; the majority identified as Cisgender female (57.7%), African American/Black (33.9%), and Hispanic/Latiné (54.8%; please see Supplementary Appendix 1 for more detailed participant characteristics). Most teens (97.1%) selected English as their preferred language, with the remaining 2.9% selecting Spanish. Of the 155 teens who opted to include parental permission, 14.8% indicated Spanish as their guardian’s preferred language (see Table 1).

**Table 1:** Participant and caregiver language preference

	*N*	%
**Teen Preferred Language (N = 239)**		
English	232	97.1%
Spanish	7	2.9%
Other	0	0.0%
**Parental Language Preference (N = 155)**		
English	132	85.2%
Spanish	23	14.8%
Language not listed	0	0.0%

Table 1 presents the language preference of both the teen participants and their guardians. Within the “other” category, teens could specify preferred languages such as Mandarin, Polish, Arabic, Hindi, Urdu, and other unlisted languages; however, no participants endorsed any of these languages

### Module 1–2: key modifications to the TeACH system

The first two modules of the FRAME-IS aim to describe the modification and identify the components of the intervention that were modified (e.g., content). We identified six key modifications made across the pre-implementation and implementation phases of the TeACH System, each of which considered the language, cultural context, and community needs of teen participants. The six key modifications were: (1) revisions to the Spanish Parental Permission Form; (2) adaptation of recruitment language used when engaging with Spanish-speaking caregivers; (3) creation of the Spanish Study Infographic; (4) training non-Spanish-speaking recruitment staff to introduce themselves in Spanish; (5) providing teens randomized to the control group with the option to receive their resources via text message or email; and (6) adapting the language of the Teen Assent Form. These modifications are presented using the FRAME-IS modules [37]. For full coding, please see Additional File 2.

### Module 3: nature of modification

Module 3 of the FRAME-IS targets the nature of the modification (e.g., adding strategies), ranging from relatively small tweaks to more sweeping changes. Most modifications (*N* = 4) were focused on tailoring/tweaking/refining study materials and recruitment language. The remaining two modifications involved adding elements to the intervention.

### Module 4

Module 4 of the FRAME-IS targets the goal of the modifications and the intended audience (i.e., participant, provider, etc.). Goals are described below for revising the Spanish Parental Permission Form, adapting recruitment language when engaging with families in Spanish, creating the Spanish Study Infographic, training non-Spanish-speaking staff to introduce themselves in Spanish, providing teens in the control group with the option to receive resources, and adapting the language of the Teen Assent Form. Following this, we note who the modifications were meant to support.

#### Goals of the modification

##### Revising the Spanish parental permission form: increase health equity

The Parental Permission Form was professionally translated verbatim, then adapted by our bilingual and bicultural research staff to ensure linguistic equivalence with the English version and revise any stigmatizing or potentially harmful language. For example, the IRB-approved English version of the Parental Permission Form described the primary risk presented by the research study as “breaches of privacy.” The verbatim Spanish translation rendered this as “violación de privacidad,” or “violation of privacy.” While this was a literal translation, the phrase did not carry the same meaning in a cultural context and was not considered culturally appropriate. Specifically, the word “violación” or “violation” carries a strong negative connotation in Spanish that can be triggering and does not align with its intended meaning in English. Our bilingual research staff identified this issue and adapted the language to “una pérdida de privacidad” or “a loss of privacy.” This change ensured that the description of the primary risk was both culturally appropriate and semantically aligned with the original English version for Spanish-speaking caregivers.

##### Avoiding stigmatizing language: increase acceptability, appropriateness, feasibility

Originally, recruitment staff described the TeACH System in Spanish as “a tool that offers resources to help teens with *feelings* of anxiety or stress” (Una herramienta que ofrece recursos para ayudar a adolescentes con *sentimientos* de ansiedad o estrés), instead of “a tool that offers resources to help teens *with* anxiety or stress” (Una herramienta que ofrece recursos para ayudar a adolescentes *con* ansiedad o estrés). Using a phrase like “*with* anxiety or stress” may discourage participation, as families might feel their teens do not fit the criteria or may be reluctant to disclose a mental health diagnosis—especially given the stigma often associated with clinical mental health conditions in the Latiné community [40–43]. By saying “*feelings* of anxiety or stress”, it normalizes experiences of anxiety and stress as they are portrayed as temporary. Understanding the cultural context of mental health of the community served is essential to reduce misunderstandings and allow families to make an informed decision about their involvement in a research study.

##### Infographic and Spanish introductions for all staff: improve fidelity and reach

As our recruitment team continued to connect with our target population at the PPC clinics, they observed a greater number of interactions with Spanish-speaking families. Since the TeACH System was an intervention designed in English and English comprehension was an inclusion criteria, it was not inherently necessary to have study materials in other languages. However, we made modifications (i.e., a Spanish Study Infographic [see Additional File 3] and training non-Spanish-speaking recruitment staff to introduce themselves in Spanish) to support the inclusion of teens with Spanish-speaking caregivers and ensure that these families received accurate information about the study.

##### Sharing resources with control group: increasing accessibility, reducing cognitive load

Teens who scored mild or greater anxiety were either randomized into the intervention group or the control group, consisting of general feedback about anxiety and a digital cognitive behavioral therapy (CBT) workbook for teens with anxiety. Initially, teens in the control group could only access their resources through the participant completion page in REDCap. Most teens were recruited using a tablet provided by study staff in clinic waiting rooms, giving them a single opportunity to save the resource link. As such, this modification is intended to reduce the cognitive load multilingual teens face when navigating access to mental health resources/support. By extending the resource-delivery options to the control group, we ensured that all interested teens had access to evidence-based tools even after their participation in the TeACH System ended.

##### Teen assent adaptation: increase reach

Through the use of a waiver of parental permission granted by the primary study site’s [MASKED] Institutional Review Board (IRB), participants had the option to continue with study activities without the involvement of a guardian or choose to have a guardian provide consent in English or Spanish. In response to feedback from teen participants during in-person recruitment about the length and complexity of the IRB-approved Teen Assent Form, we revised the language to significantly reduce research jargon and make the form more concise. Given that a waiver of parental permission was obtained for this trial, it was essential that *all* teen participants clearly understood what their participation in the TeACH System entailed to provide informed consent. Similarly, the assent form was also adapted to make participation in the TeACH System more accessible for teens from multilingual families. We noted that teens from multilingual families may not have a caregiver who can effectively support them in comprehending English documentation or research jargon [44]. To address this challenge, it was necessary to revise the assent form with more teen-friendly language to foster better engagement and informed consent.

#### Audience of the modification

The audience for the modifications was either at the patient level (e.g., creating the Spanish Study Infographic and adapting the recruitment language into Spanish; 83.3%) or at the implementor level (e.g., training English-speaking staff to introduce themselves in Spanish; 16.6%).

### Module 5: when were the modifications made & were they planned

One modification was made during the pre-implementation phase (i.e., revisions to the Spanish Parental Permission Form), and the remaining five modifications to the TeACH system were made during the implementation phase (83.3%). Only the revisions to the Spanish Parental Permission Form and training English-speaking recruitment staff to introduce themselves in Spanish were planned/proactive modifications. All other modifications were unplanned/reactive (66.7%).

### Module 6–7: who participated in the decision to modify & how widespread were the modifications

Recruitment staff (i.e., postdoctoral fellow, research associates, undergraduate volunteers) led the majority (83.3%) of decisions regarding modifications. The decision to give teens in the control group the option to receive their resources via text message or email involved the principal investigator. The teen feedback was a key driver in the decision to adapt the Teen Assent Form. Regarding the breadth and scope of the modifications, all modifications were made to improve the TeACH System and better serve multilingual teens and families.

## Discussion

The present study aims to describe modifications, informed by feedback from key players (i.e., recruitment staff and research participants), to a monolingual D-LIT implemented in urban primary care to increase access to evidence-based resources for multilingual families. Even though the TeACH System was originally developed in English for English-speaking teens, iterative refinements to participant-facing materials, recruitment procedures, and communication strategies were essential for ensuring accessibility for teens from multilingual families. Our findings highlight the central role of *culturally-responsive* modifications in enhancing the equity of D-LITs in real-world settings. While most modifications were unplanned, they were not random—they emerged in response to input from key players and a commitment to equitable, culturally-responsive care.

First, culturally-responsive modifications prompt researchers to consider the ecological context in which interventions are delivered. Even when the intervention is intended to be delivered in English, implementation processes must account for the linguistic needs of participants’ families. While D-LITs offer individuals resources to self-manage symptoms, it is important to make implementation procedures adaptable to better engage multilingual families. Teens from multilingual families are often placed in the role of ad hoc interpreters for their caregivers—especially in healthcare settings—leading to reduced support and increased emotional burden (e.g., depressive symptoms) [44, 45]. Modifications, such as revising the Spanish Parental Permission Form and developing the Spanish Study Infographic, empower teens to seek out support from their caregivers without the additional burden of translation and enable caregivers with limited English proficiency to participate in their teens’ access to a mental health tool.

Next, verbatim translations are insufficient. Translating participant-facing materials is a basic responsibility to ensure that information is communicated clearly and accurately to all participants, regardless of their preferred language. However, it does not guarantee linguistic or cultural appropriateness. Direct, verbatim translation may inadvertently introduce stigmatizing or confusing terminology—particularly in a mental health context, where nuance is critical. The miscommunication identified in the Spanish Parental Permission Form (i.e., “violación de privacidad”) underscores the importance of supplementing professional translation with review by a community member who understands the linguistic and cultural connotations of key terms. Culturally sensitive wording helped mitigate potential misunderstandings or stigma surrounding anxiety and research participation. As such, the community’s cultural understanding of mental health may help inform appropriate engagement strategies to better reach multilingual families.

Finally, iterative feedback from key players, such as research participants and recruitment staff, is essential. A defining characteristic of effective implementation is its responsiveness to context. As illustrated by this study, 83.3% of the modifications were prompted by observations from the recruitment team and direct feedback from teen participants. The majority of modifications (66.7%) were unplanned and difficult to anticipate—highlighting the importance of flexible, responsive implementation strategies. The reality is that in dynamic, real-world settings—such as PPC clinics—unforeseen challenges will arise. Rather than viewing modifications as a deviation from the protocol, our findings support the view that modifications are a necessary and ongoing part of equitable implementation.

### Implications of modifications for equity

Our study directly responds to the growing opportunity for action in implementation science to center equity in the development and dissemination of EBTs. The monolingual approach to developing, testing, and deploying D-LITs—and DMH tools broadly—primarily in English often excludes multilingual families. This approach increases the risk of perpetuating inequities in access to mental healthcare [46]. Actively incorporating feedback from research participants and recruitment staff—many of whom reflect the cultural and linguistic diversity of the target population—can inform culturally-responsive modifications. It is imperative that DMH tools be designed and modified in ways that do not exacerbate existing disparities, as healthcare increasingly relies on the potential of D-LITs and various DMH tools to offer accessible, scalable EBTs [46]. The TeACH System is an English-language, self-guided D-LIT; yet our findings underscore that even in English-delivered interventions, supplemental materials and implementation strategies (e.g., Spanish Study Infographic, revised Spanish Parental Permission forms) are critical to ensuring equitable access. Without these modifications, teens from multilingual families would have been denied the opportunity to involve their caregivers, with about 46.1% of youth opting for caregiver involvement, and a proportion of caregivers being Spanish-speaking (14.8%). Our findings underscore that modifications made during design and implementation are not only the norm but also critical for equitable care, ensuring that DMH tools evolve to meet the needs of diverse populations.

### Future directions

As DMH continues to expand globally, researchers should prioritize systematic documentation and evaluation of modifications that center equity. While it was not documented in the Results as a modification made during pre-implementation or implementation, the use of a waiver of parental permission for a teen-focused DMH trial is relatively novel and may have supported increased teen engagement. Future research should explore the role of waivers of guardian permission in engagement among teens from linguistically and culturally diverse communities, particularly when they may help teens access care that their guardians may view as stigmatizing. Building on this study, future efforts might examine how culturally-responsive modifications impact engagement, clinical outcomes, and the sustainability of D-LITs among multilingual families.

### Limitations

This study is limited in its scope and generalizability. The modifications described were based on a specific implementation context—PPC clinics in Chicago, IL, USA—which may not fully capture the needs of multilingual families in other geographic or cultural contexts. Moreover, the research team did not assess the impact of these modifications on treatment outcomes or engagement. While the FRAME-IS framework provided systematic documentation of the modifications, future studies should examine how specific cultural modifications influence health outcomes and engagement.

## Conclusion

Our study affirms the notion that fidelity and fit are not mutually exclusive; in fact, culturally-responsive modifications are essential to enhancing the accessibility and equity of evidence-based practices. This is especially true for D-LITs like the TeACH System, which are rapidly expanding across diverse, real-world contexts. Although the modifications described here were developed for a PPC setting serving teens with Spanish-speaking caregivers, the implications extend broadly to the implementation of DMH tools for culturally and linguistically diverse families. By systematically documenting modifications using a framework such as FRAME-IS, researchers can promote transparency and replicability while advancing equity-driven implementation strategies. Ultimately, this work underscores a critical responsibility: not only to design clinically effective interventions but also to refine and deliver them in ways that center the needs, languages, and lived experiences of the communities they aim to serve.

## Supplementary Information

Below is the link to the electronic supplementary material.

Supplementary Material 1

Supplementary Material 2

Supplementary Material 3

## Data Availability

The datasets used and/or analysed during the current study are available from the corresponding author on reasonable request.

## Abbreviations

DMH: Digital Mental Health Tools
PPC: Pediatric Primary Care
FRAME-IS: The Framework for Reporting Adaptations and Modifications to Evidence-based Implementation Strategies
TeACH: The Teen Assess, Check, and Heal System
D-LITs: Digital Low-Intensity Treatments

## References

[1] McGorry PD, Mei C, Dalal N, Alvarez-Jimenez M, Blakemore SJ, Browne V, et al. The Lancet Psychiatry Commission on youth mental health. Lancet Psychiatry. 2024;11(9):731–74. 10.1016/S2215-0366(24)00163-9. 39147461

[2] McGorry P, Gunasiri H, Mei C, Rice S, Gao CX. The youth mental health crisis: analysis and solutions. Front Psychiatry. 2025;15:1517533. 10.3389/fpsyt.2024.1517533. PubMed PMID: 39906686; PubMed Central PMCID: PMC11790661. PMC11790661 39906686

[3] CDC. Youth Risk Behavior Surveillance System (YRBSS) [Internet]. 2025 [cited 2025 Jul 6]. YRBS Data Summary & Trends Report. Available from: https://www.cdc.gov/yrbs/dstr/index.html.

[4] Office of the Surgeon General (OSG). Protecting youth mental health: The U.S. surgeon general’s advisory [Internet]. Washington (DC): US Department of Health and Human Services; 2021 [cited 2025 Jul 6]. (Publications and Reports of the Surgeon General). Available from: http://www.ncbi.nlm.nih.gov/books/NBK575984/. PubMed PMID: 34982518. 34982518

[5] O’Brien D, Harvey K, Howse J, Reardon T, Creswell C. Barriers to managing child and adolescent mental health problems: a systematic review of primary care practitioners’ perceptions. Br J Gen Pract. 2016;66(651):e693–707. 10.3399/bjgp16X.687061. PubMed PMID: 27621291; PubMed Central PMCID: PMC5033306. PMC5033306 27621291

[6] Salloum A, Johnco C, Lewin AB, McBride NM, Storch EA. Barriers to access and participation in community mental health treatment for anxious children. J Affect Disord. 2016;196:54–61. 10.1016/j.jad.2016.02.026. 26901657

[7] Kirkbride JB, Anglin DM, Colman I, Dykxhoorn J, Jones PB, Patalay P, et al. The social determinants of mental health and disorder: evidence, prevention and recommendations. World Psychiatry. 2024;23(1):58–90. 10.1002/wps.21160. PubMed PMID: 38214615; PubMed Central PMCID: PMC10786006. PMC10786006 38214615

[8] Alegría M, Xiong M, Sánchez González ML. The Role of Social Determinants in Racial and Ethnic Mental Health Disparities: Getting It Right. Harv Rev Psychiatry. 2025;33(2):67. 10.1097/HRP.0000000000000421. 40036024

[9] S1601: language spoken at home. - Census Bureau Table [Internet]. [cited 2025 Jul 28]. Available from: https://data.census.gov/table/ACSST5Y2020.S1601?t=Language+Spoken+at+Home&g=010XX00US&y=2020.

[10] B16004: age by language spoken at. … Census Bureau Table [Internet]. [cited 2025 Jul 28]. Available from: https://data.census.gov/table/ACSDT5Y2020.B16004?t=Language+Spoken+at+Home&g=010XX00US&y=2020.

[11] Duong LT, Tran MA. Caregivers with limited English proficiency: satisfaction with primary pediatric healthcare. J Child Health Care Prof Work Child Hosp Community. 2024;13674935241252479. 10.1177/13674935241252479. PubMed PMID: 38718378. PMC12662825 38718378

[12] Roller M, Snow K, Grapa W, Hernández J, Consolí M, Pineda A. Estimating the Impact of Limited English Language Proficiency on Mental Health Services for Spanish Speakers in the United States. J Natl Hisp Med Assoc. 2023;1(2):10–7. 10.59867/nhma0202.

[13] DP02. Selected Social … Census Bureau Table [Internet]. [cited 2025 Jul 12]. Available from: https://data.census.gov/table/ACSDP5Y2023.DP02.

[14] Quinteros Baumgart C, Billick SB. Positive Cognitive Effects of Bilingualism and Multilingualism on Cerebral Function: a Review. Psychiatr Q. 2018;89(2):273–83. 10.1007/s11126-017-9532-9. 28895004

[15] Banda F, James G, Vasudeva K, Franklin M, Thoumi A, Cholera R. Building Equitable Mental Health Care for Latino Children: Perspectives from Providers and Communities. J Racial Ethn Health Disparities. 2025;12(3):1598–611. 10.1007/s40615-024-01992-4. PMC11745160 38592661

[16] Bauer AM, Alegría M. Impact of Patient Language Proficiency and Interpreter Service Use on the Quality of Psychiatric Care: A Systematic Review. Psychiatr Serv. 2010;61(8):765–73. 10.1176/ps.2010.61.8.765. PMC2946248 20675834

[17] Greenhalgh T. Case studies: a guide for researchers, educators, and implementers. BMJ Med. 2025;4(1):e001623. 10.1136. /bmjmed-2025-001623 PubMed PMID: 41000468; PubMed Central PMCID: PMC12458855. 10.1136/bmjmed-2025-001623 PMC12458855 41000468

[18] Lorenzo-Luaces L, Fite RE. Wider, faster, more: Reenvisioning depression treatment research in the United States. Pract Innov. 2024;9(1):19–29. 10.1037/pri0000240.

[19] Cuijpers and Schuurmans PiP. Self-help interventions for anxiety disorders: an overview. [Internet]. [cited 2025 Jun 16]. Available from: https://core.ac.uk/reader/15456048?utm_source=linkout. 10.1007/s11920-007-0034-6 17880859

[20] Petrovic M, Gaggioli A. Digital mental health tools for caregivers of older adults—a scoping review. Front Public Health. 2020;8. 10.3389/fpubh.2020.00128. PMC7198719 32411643

[21] Sijbrandij M, Kleiboer A, Farooq S, Editorial. Low-Intensity Interventions for Psychiatric Disorders. Front Psychiatry. 2020;11:619871. 10.3389/fpsyt.2020.619871. PubMed PMID: 33324269; PubMed Central PMCID: PMC7723924. PMC7723924 33324269

[22] Object object. Self-help interventions for anxiety disorders: An overview. [Internet]. [cited 2025 Jun 16]. Available from: https://core.ac.uk/reader/15456048?utm_source=linkout.

[23] Suen YN, Leung CM, Wan YS, Chan KT, Wong MTH, Hui CLM, et al. Evaluation of a Low-Intensity Online Intervention (LiON) for Reducing Distress and Mental Health Symptoms in Young People. J Adolesc Health Off Publ Soc Adolesc Med. 2024;74(1):89–97. 10.1016/j.jadohealth.2023.08.022. PubMed PMID: 37815770. 37815770

[24] Arean PA, Hallgren KA, Jordan JT, Gazzaley A, Atkins DC, Heagerty PJ, et al. The Use and Effectiveness of Mobile Apps for Depression: Results From a Fully Remote Clinical Trial. J Med Internet Res. 2016;18(12):e6482. 10.2196/jmir.6482. PMC5209607 27998876

[25] Stiles-Shields C, Bobadilla G, Reyes K, Gustafson EL, Lowther M, Smith DL, et al. Digital Mental Health Screening, Feedback, and Referral System for Teens With Socially Complex Needs: Protocol for a Randomized Controlled Trial Integrating the Teen Assess, Check, and Heal System into Pediatric Primary Care. JMIR Res Protoc. 2025;14(1):e65245. 10.2196/65245. PMC11888113 39965196

[26] Ansell DA, Oliver-Hightower D, Goodman LJ, Lateef OB, Johnson TJ. Health equity as a system strategy: the Rush University Medical Center framework. NEJM Catal. 2021;2(5). 10.1056/CAT.20.0674.

[27] 2020 Census Hispanic or Latino Population Data | Great Cities Institute [Internet]. [cited 2025 Jun 16]. Available from: https://greatcities.uic.edu/2021/11/24/2020-census-hispanic-or-latino-population-data/.

[28] Chicago city. Illinois - Census Bureau Profile [Internet]. [cited 2025 Jun 16]. Available from: https://data.census.gov/profile/Chicago_city,_Illinois?g=160XX00US1714000

[29] Stiles-Shields C, Gustafson EL, Lim PS, Bobadilla G, Thorpe D, Summersett Williams FC, et al. Pre-implementation determinants for digital mental health integration in Chicago pediatric primary care. J Pediatr Psychol. 2025;50(1):86–95. 10.1093/jpepsy/jsae058. PMC11753843 39101560

[30] Wiltsey Stirman S, Gamarra JM, Bartlett BA, Calloway A, Gutner CA. Empirical Examinations of Modifications and Adaptations to Evidence-Based Psychotherapies: Methodologies, Impact, and Future Directions. Clin Psychol Sci Pract. 2017;24(4):396–420. 10.1111/cpsp.12218. PMC5866913 29593372

[31] Stirman SW, Miller CJ, Toder K, Calloway A. Development of a framework and coding system for modifications and adaptations of evidence-based interventions. Implement Sci. 2013;8(1):65. 10.1186/1748-5908-8-65. PMC3686699 23758995

[32] Wiltsey Stirman S, Gutner A, Crits-Christoph C, Edmunds P, Evans J, Beidas AC. Relationships between clinician-level attributes and fidelity-consistent and fidelity-inconsistent modifications to an evidence-based psychotherapy. Implement Sci. 2015;10(1):115. 10.1186/s13012-015-0308-z. PMC4534152 26268633

[33] Chambers DA, Norton WE. The Adaptome: Advancing the Science of Intervention Adaptation. Am J Prev Med. 2016;51(4 Suppl 2):S124–131. 10.1016/j.amepre.2016.05.011. PubMed PMID: 27371105; PubMed Central PMCID: PMC5030159. PMC5030159 27371105

[34] Baumann AA, Cabassa LJ. Reframing implementation science to address inequities in healthcare delivery. BMC Health Serv Res. 2020;20(1):190. 10.1186/s12913-020-4975-3. PubMed PMID: 32164706; PubMed Central PMCID: PMC7069050. PMC7069050 32164706

[35] Morris ZS, Wooding S, Grant J. The answer is 17 years, what is the question: understanding time lags in translational research. J R Soc Med. 2011;104(12):510–20. 10.1258/jrsm.2011.110180. PubMed PMID: 22179294; PubMed Central PMCID: PMC3241518. PMC3241518 22179294

[36] Bobadilla G, Gustafson EL, Stiles-Shields C. Commentary: We don’t know what we don’t know: co-design and implementation science to advance digital psychological interventions. J Pediatr Psychol. 2026;jsag026. 10.1093/jpepsy/jsag026. PMC13626090 41974111

[37] Miller CJ, Barnett ML, Baumann AA, Gutner CA, Wiltsey-Stirman S. The FRAME-IS: a framework for documenting modifications to implementation strategies in healthcare. Implement Sci. 2021;16(1):36. 10.1186/s13012-021-01105-3. PMC8024675 33827716

[38] Rezigalla AA. Observational Study Designs: Synopsis for Selecting an Appropriate Study Design. Cureus. 2020;12(1):e6692. 10.7759/cureus.6692. PubMed PMID: 31988824; PubMed Central PMCID: PMC6970097. PMC6970097 31988824

[39] Schoenthaler A, De La Calle F, De Leon E, Garcia M, Colella D, Nay J, et al. Application of the FRAME-IS to a multifaceted implementation strategy. BMC Health Serv Res. 2024;24:695. 10.1186/s12913-024-11139-0. PubMed PMID: 38822342; PubMed Central PMCID: PMC11143702. PMC11143702 38822342

[40] Grieb SM, Platt R, Vazquez MG, Alvarez K, Polk S. Mental health stigma among Spanish-speaking Latinos in Baltimore, Maryland. J Immigr Minor Health. 2023;1–9. 10.1007/s10903-023-01488-z. PubMed PMID: 37213041; PubMed Central PMCID: PMC10201042. PMC10201042 37213041

[41] Ahmedani BK. Mental Health Stigma: Society, Individuals, and the Profession. J Soc Work Values Ethics. 2011;8(2):4–1. PubMed PMID: 22211117; PubMed Central PMCID: PMC3248273. PMC3248273 22211117

[42] Gearing RE, Washburn M, Brewer KB, Cabrera A, Yu M, Torres LR. Pathways to mental health care: Latinos’ help-seeking preferences. J Latinx Psychol. 2024;12(1):1–17. 10.1037/lat0000237.

[43] Caplan S. Intersection of Cultural and Religious Beliefs About Mental Health: Latinos in the Faith-Based Setting. Hisp Health Care Int Off J Natl Assoc Hisp Nurses. 2019;17(1):4–10. 10.1177/1540415319828265. PubMed PMID: 30799650. 30799650

[44] Shen Y, Seo E, Jiles AI, Zheng Y, Wang Y. Language brokering and immigrant-origin youth’s well-being: A meta-analytic review - ProQuest [Internet]. 2022 [cited 2025 Jul 17]. Available from: https://www.proquest.com/docview/2720485041?accountid=uic&pq-origsite=primo&sourcetype=Scholarly%20Journals#REF_c48 10.1037/amp0001035 36190758

[45] Kam JA, Lazarevic V. The Stressful (and Not So Stressful) Nature of Language Brokering: Identifying When Brokering Functions as a Cultural Stressor for Latino Immigrant Children in Early Adolescence. J Youth Adolesc. 2014;43(12):1994–2011. 10.1007/s10964-013-0061-z. 24241786

[46] Baumann AA, Shelton RC, Kumanyika S, Haire-Joshu D. Advancing healthcare equity through dissemination and implementation science. Health Serv Res. 2023;58(Suppl 3):327–44. 10.1111/1475-6773.14175. PubMed PMID: 37219339; PubMed Central PMCID: PMC10684051. PMC10684051 37219339

