# Supplementary material for: More than translation: an exploratory case study of culturally-responsive modifications in digital mental health for multilingual families
Source: BMC Health Serv Res. 2026 Jul 30;26:1493. doi: 10.1186/s12913-026-15220-8 (PMC13726146; doi:10.1186/s12913-026-15220-8)
Supplement: Supplementary file 1 [file 12913_2026_15220_MOESM1_ESM.pdf]

Supplementary Table 1. Participant Demographics (n= 239)

	N	%	*M*
**Age (years)**			15.20
**Ethnicity, n (%)**			
Hispanic/Latine	131	54.8%	
MENA (Middle Eastern/North African)	11	4.6%	
None of the Above	82	34.3%	
Prefer Not to Answer	10	4.2%	
**Race, n (%)**			
African American/Black	81	33.9%	
American Indian or Alaska Native	10	4.2%	
Asian	8	3.3%	
Native Hawaiian or Other Pacific Islander	4	1.7%	
White	73	40.5%	
Prefer Not to Answer	60	25.1%	
**Gender Identity, n (%)**			
Cisgender Female	138	53.7%	
Cisgender Male	75	31.4%	
Transgender Female	0	0.0%	
Transgender Male	4	1.7%	
Non-Binary	1	0.4%	
Option Not Listed	8	3.4%	
Prefer Not to Answer	10	4.2%	

Supplementary Table 1 presents detailed participant demographics from the TeACH System Trial.
