# Supplementary material for: More than translation: an exploratory case study of culturally-responsive modifications in digital mental health for multilingual families
Source: BMC Health Serv Res. 2026 Jul 30;26:1493. doi: 10.1186/s12913-026-15220-8 (PMC13726146; doi:10.1186/s12913-026-15220-8)
Supplement: Supplementary file 2 [file 12913_2026_15220_MOESM2_ESM.pdf]

Additional File 1. FRAME-IS Coding for Key Modifications

	Module 1		Module 2	Module 3	Module 4, Part 1	Module 4, Part 2	Module 5, Part 1	Module 5, Part 2	Module 6	Module 7
	The modification(s) being made is/are:	The reason(s) for the modification(s) is/are:	WHAT is modified?	What is the NATURE of the content, evaluation, or training modification?	What is the GOAL?	What is the LEVEL of the rationale for modification?	WHEN is the modification initiated?	Is modification PLANNED?	WHO participates in the decision to modify?	How WIDESPREAD is the modification? (i.e., for whom/what is the modification made?
1	Revisions to the Spanish Parental Permission Form	To ensure linguistic equivalence with the English version and revise any stigmatizing or potentially harmful language	Content	Tailoring/tweaking/refining	Increase health equity or decrease disparities in EBP delivery	Patient or Other Receipient level (i.e. those who will ideally benefit from the EBP)	Pre-implementation/planning/pilot phase	Planned/Proactive	Implementer or implementation strategy expert	Patients or other recipients that share a particular characteristic (e.g. all patients from a specific language background)
2	Adapting recruitment language when engaging with Spanish-speaking families	To avoid stigmatizing language around mental health.	Training	Tailoring/tweaking/refining	Increase the acceptability, appropriateness, or feasibility of the implementation effort (i.e. improve the fit between the implementation effort and the needs of those delivering the EBP)	Patient or Other Receipient level (i.e. those who will ideally benefit from the EBP)	Implementation phase	Unplanned/Reactive	Implementer or implementation strategy expert	Patients or other recipients that share a particular characteristic (e.g. all patients from a specific language background)
3	Create a Spanish Infographic that details the study's objectives and procedures.	To provide accurate and transparent study information when non-Spanish-speaking staff encounter Spanish-speaking caregivers during in-person recruitment	Content	Adding elements	Improve fidelity to the EBP (i.e. improve the extent to which the EBP is delivered as intended)	Patient or Other Receipient level (i.e. those who will ideally benefit from the EBP)	Implementation phase	Unplanned/Reactive	Implementer or implementation strategy expert	Patients or other recipients that share a particular characteristic (e.g. all patients from a specific language background)
4	Training non-Spanish-speaking recruitment staff to introduce themselves in Spanish	To support the inclusion and engagement of teens with Spanish-speaking caregivers	Training	Tailoring/tweaking/refining	Increase the acceptability, appropriateness, or feasibility of the implementation effort (i.e. improve the fit between the implementation effort and the needs of those delivering the EBP)	Implementer level (i.e. those charged with leading the implementation effort)	Implementation phase	Planned/Proactive	Implementer or implementation strategy expert	Patients or other recipients that share a particular characteristic (e.g. all patients from a specific language background)
5	Providing teens randomized to the control group with the option to receive their resources via text message or email	To ensure that all interested teens have access to evidence-based tools even after their participation concludes with the TeACH System. Also, to ease the cognitive load of multilingual teens navigating the acquisition of tools that may be beneficial to their mental health and well-being.	Evaluation	Adding elements	Improve sustainability of the EBP (i.e. increase the chances that the EBP remains in practice after the implementation effort ends)	Patient or Other Receipient level (i.e. those who will ideally benefit from the EBP)	Implementation phase	Unplanned/Reactive	Researcher, Implementer or implementation strategy expert	Patients or other recipients that share a particular characteristic (e.g. all patients from a specific language background)
6	Adapting the language of the Teen Assent Form	To use more approachable language, helping all teen participants clearly understand their involvement in the research study, especially teens from multilingual families who may not have a caregiver who can effectively support them in comprehending documentation or jargon in English	Content	Tailoring/tweaking/refining	Increase reach of the EBP (i.e. the # of patients receiving the EBP)	Patient or Other Receipient level (i.e. those who will ideally benefit from the EBP)	Implementation phase	Planned/Reactive	Patients or other receipients who will be the ultimate target	Patients or other recipients that share a particular characteristic (e.g. all patients from a specific language background)

Additional File 1 presents the data set for this exploratory case study with each modification systematically documented using the FRAME-IS.
