# Supplementary material for: More than translation: an exploratory case study of culturally-responsive modifications in digital mental health for multilingual families
Source: BMC Health Serv Res. 2026 Jul 30;26:1493. doi: 10.1186/s12913-026-15220-8 (PMC13726146; doi:10.1186/s12913-026-15220-8)
Supplement: Supplementary file 3 [file 12913_2026_15220_MOESM3_ESM.pdf]

Additional File 3. Modification 3 Spanish Study Infographic

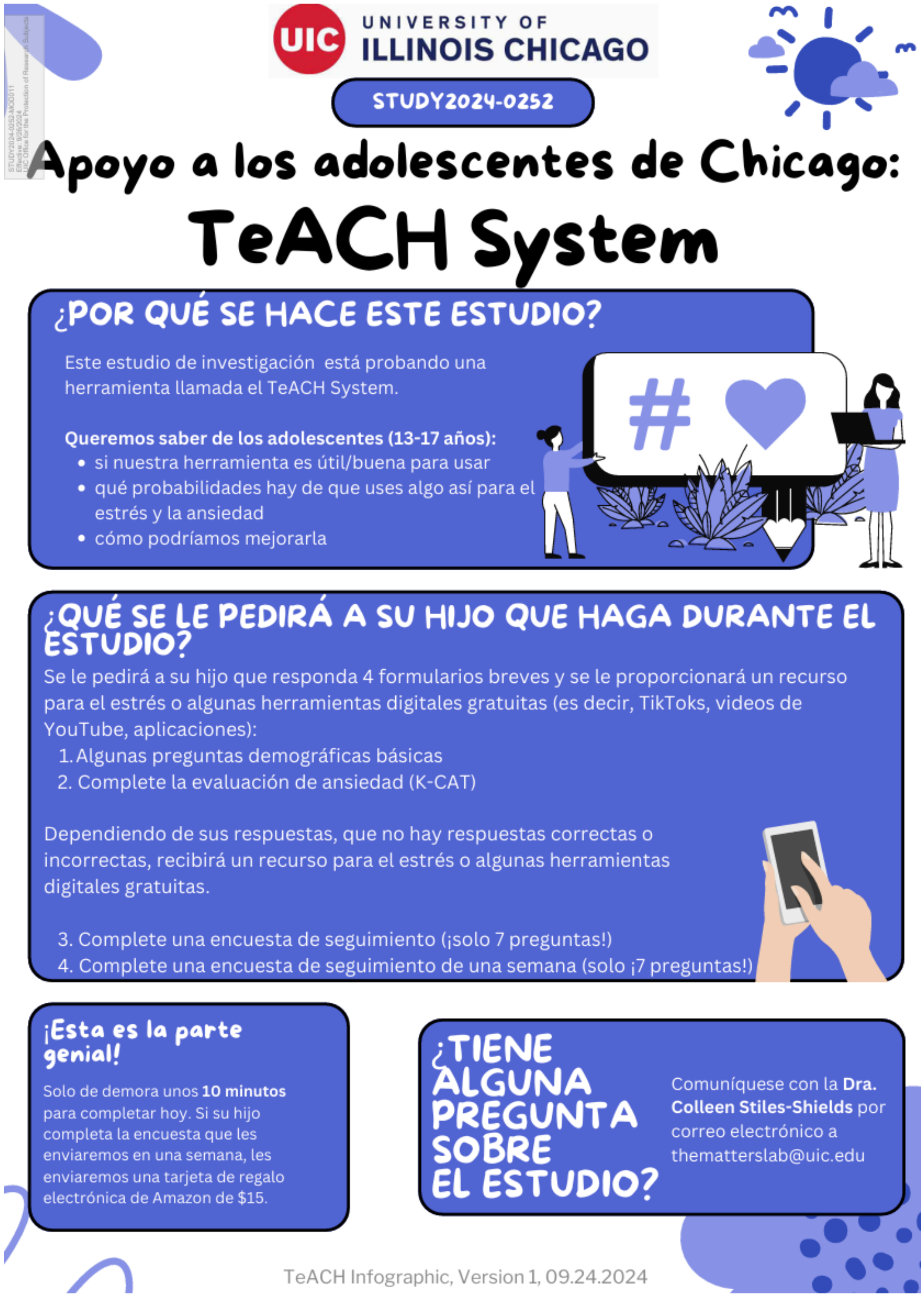

Additional File 3 presents the Spanish Study Infographic described in modification 3 to provide Spanish-speaking caregivers with accurate information about the TeACH System Study. The Spanish Study Infographic supported non-Spanish speaking staff in interacting with Spanish-speaking families during recruitment.
